# A Q_slope_-based empirical method to stability assessment of mountain rock slopes in multiple faults zone: A case for North of Tabriz

**DOI:** 10.1016/j.mex.2022.101718

**Published:** 2022-04-29

**Authors:** Mehdi Kouhdaragh, Mohammad Azarafza, Reza Derakhshani

**Affiliations:** a Department of Civil Engineering, Malekan Branch, Islamic Azad University, Malekan, Iran; b Department of Civil Engineering, University of Tabriz, Tabriz, Iran; c Department of Geology, Shahid Bahonar University of Kerman, Iran; d Department of Earth Sciences, Utrecht University, Utrecht, Netherlands

**Keywords:** Rock slope engineering, Slope stability, Rock slope classification, Q_slope_, Fault zone

## Abstract

The present article provides an empirical relationship for rock slope stability assessment based on Q_slope_ classification. The relationship is used as a correction procedure for classic Q_slope_ for mountain regions with multiple fractures related to several faults. The relationship is derived from 25 distinct jointed slopes near the North Tabriz Fault (NTF). The NTF triggered numerous micro-faults and fractures in rocky landscapes, resulting in sliding on a variety of scales. The present empirical method is introduced based on a field survey and a stability analysis of the studied slopes based on Q_slope_ principles. The results indicate that the classic formulation of Q_slope_ can be modified to β = 62.6 log10 (Q_slope_) + 36 for mountain regions with multiple fault zones.

• This empirical method can be useful for fast stability assessment on jointed rock slopes.

• This relationship can use as a modification for the original formula in multiple faults zones.

## Graphical Abstract

**Figure:**
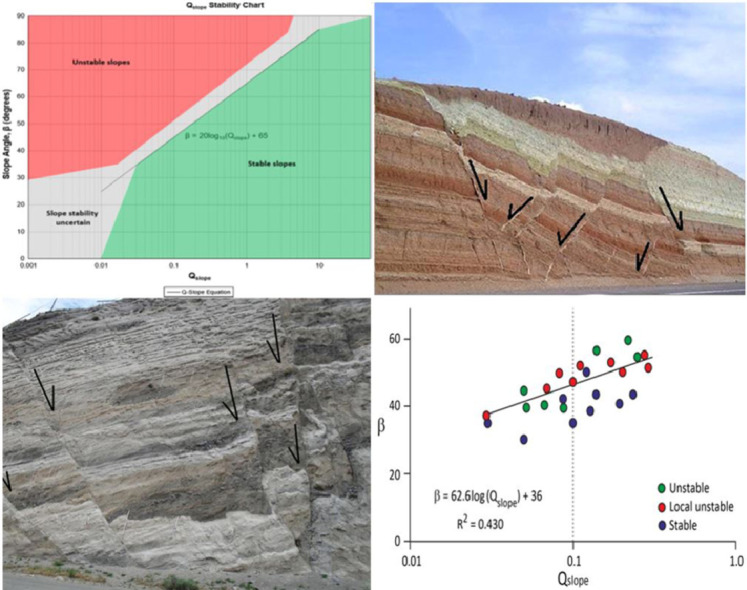

Specifications table

**Table:** 

Subject Area:	*Engineering / Slope Stability*
More specific subject area:	*Geotechnical Engineering*
Method name:	*Q_slope_ for multiple faults zone*
Name and reference of original method:	*Bar, N., Barton, N., 2017. The Q-slope method for rock slope engineering, Rock Mechanics and Rock Engineering, 50, 3307–3322.*https://doi.org/10.1007/s00603-017-1305-0. *Azarafza, M., Nanehkaran, Y.A., Rajabion, L., Akgün, H., Rahnamarad, J., Derakhshani, R., Raoof, A., 2020. Application of the modified Q-slope classification system for sedimentary rock slope stability assessment in Iran, Eng. Geol. 264, 105349.*https://doi.org/10.1016/j.enggeo.2019.105349.
Resource availability:	*There are no special resources and field investigation data is presented within the article.*

## Method details

The Q_slope_ method was originally developed by Bar and Barton [1] based on the regular Q-system classification [2]. The classification system obtained an empirical regression between the slope surface angle (β) and Q_slope_ number. This number is estimated based on several considerations and regulations which involve rock block size, geometrical condition of discontinuity network, shear force element of rock mass, external loading, and in-situ stress factor [3], [4], [5]. Bar and Barton [1] present formulations for the Q_slope_ number and β calculations as shown in Eq. (1) and (2) where RQD is the rock quality designation, J_n_ is the number of the discontinuity set, J_r_ is the discontinuity roughness number, J_a_ is discontinuity alteration number, J_wice_ is the environmental conditions number, and SRF is the slope-relevant strength reduction factor [1].

(1) $$Q_{\text{slope}}=\left(\frac{RQD}{J_{n}}\right)\times {\left(\frac{J_{r}}{J_{a}}\right)}_{0}\times \left(\frac{J_{vice}}{SRF_{slope}}\right)$$

(2) $$\beta =20\log_{10}\left[Q_{slope}\right]+65$$

This formulation is used to calculate the empirical steepest slope angle (β), which was originally proposed by Barton and Bar [5] and modified by Azarafza et al. [3]. These researchers develop stability charts for quick evaluations and determine slope reliability. The values of the β need to be stabilized through the use of slope reinforcement techniques in complex geological conditions such as frozen ground and weathered geo-units [6], [7], [8]. The current study attempted to estimate the value of the β relationship for slope stability assessments in mountain regions with multiple fault zones that affect the slope stability.

**Fig. 1:**
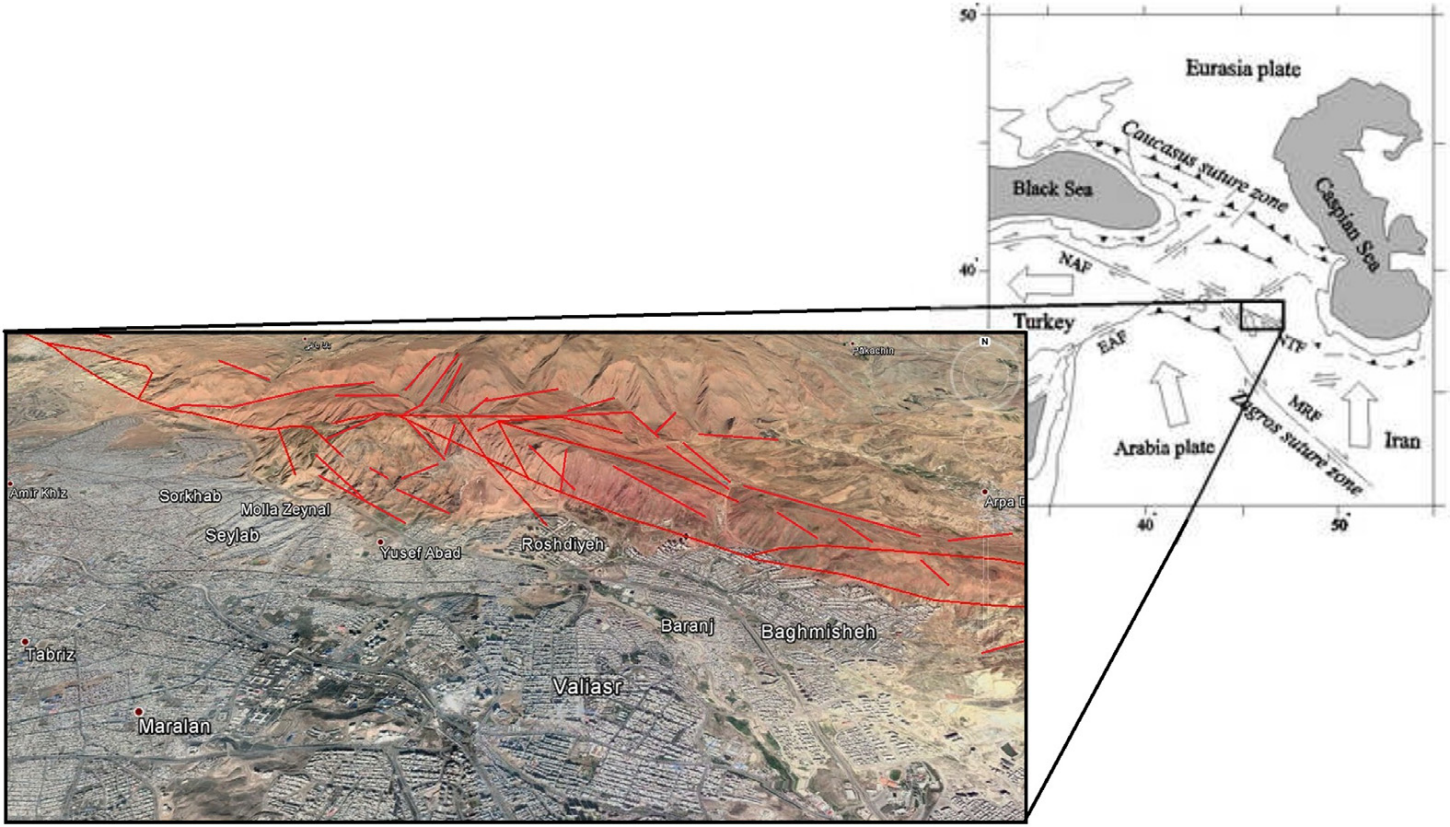
The location of the NTF zone in Iran and Tabriz [11,12].

**Fig. 2:**
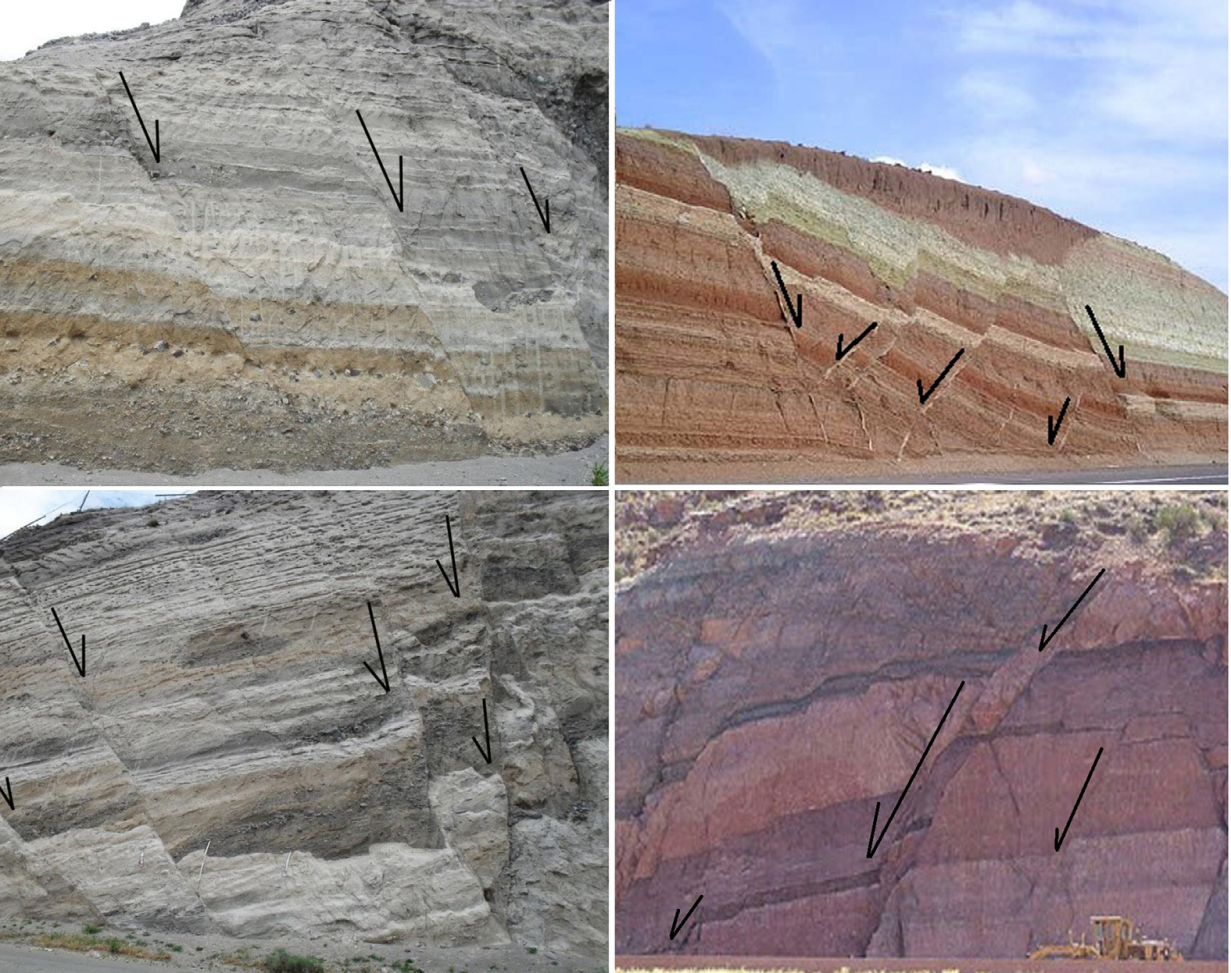
A view of the various slopes that were investigated.

The studied cases are selected from the north part of Tabriz city, located in the northwest of Iran. The North Tabriz fault (NTF) is a multi-scale fault zone that affects the geomorphology of the north part of the city and could be considered active due to several seismic activities and geological deformations throughout the region [9,10]. The NTF is located north of Tabriz, as illustrated in Fig. 1, can be effective in triggering other faults/fractures in the region [11] as well as the formation of complex geostructural landforms in the area and north of Tabriz [12]. Fig. 2 illustrates several slopes beneath numerous faults in the studied area.

**Fig. 3:**
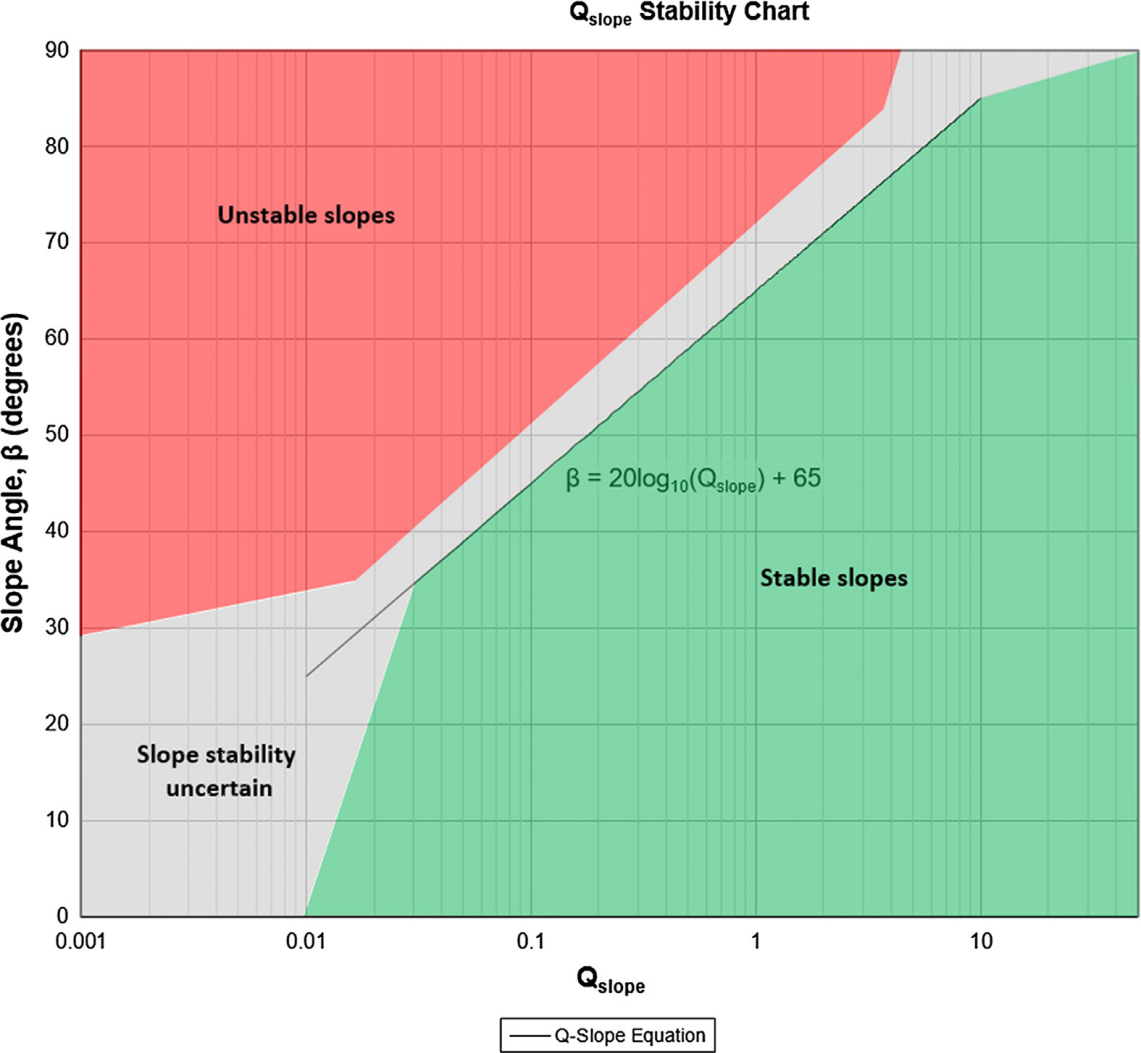
The Q_slope_ stability chart [1,3].

**Fig. 4:**
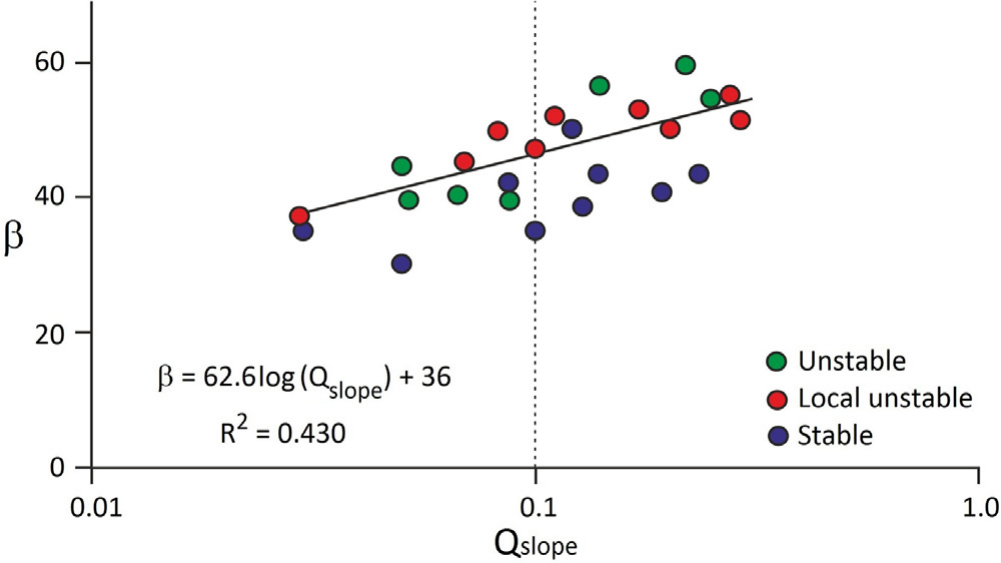
The stability chart for Q_slope_ in multi fault zones.

For stability assessment, 25 cases are used for Q_slope_-based analysis. The required data is recorded during the field survey [13,14]. A relationship is given for multiple fault zones based on the Q_slope_ classic method. Fig. 3 provides information about the studied slopes based on the stability chart [1,3]. According to the figure, there are some differences between the results and the graphic chart. Fig. 4 provides the modified chart for the stability condition of the studied slopes. By considering Fig. 2, the steepest slope angle or β was estimated. Based on the regression analysis conducted on the studied slopes’ data, it has appeared that the β can be modified as Eq. (3).

(3) $$\beta =62.6\;\log_{10}\left[Q_{slope}\right]+36$$

By employing this equation, one can gain a better understanding of the fault effects in Q_slope_ in a mountain area with a complex geological history.
